# Comparison of *Thermobifida fusca* Cellulases Expressed in *Escherichia coli* and *Nicotiana tabacum* Indicates Advantages of the Plant System for the Expression of Bacterial Cellulases

**DOI:** 10.3389/fpls.2015.01047

**Published:** 2015-12-01

**Authors:** Johannes Klinger, Rainer Fischer, Ulrich Commandeur

**Affiliations:** ^1^Institute for Biology VII (Molecular Biotechnology), RWTH Aachen UniversityAachen, Germany; ^2^Fraunhofer Institute for Molecular Biology and Applied EcologyAachen, Germany

**Keywords:** recombinant cellulases, transient plant expression, bacterial expression, *Escherichia coli*, *Nicotiana tabacum*

## Abstract

The economic conversion of lignocellulosic biomass to biofuels requires in addition to pretreatment techniques access to large quantities of inexpensive cellulases to be competitive with established first generation processes. A solution to this problem could be achieved by plant based expression of these enzymes. We expressed the complete set of six cellulases and an additional β-glucosidase expressed from *Thermobifida fusca* in the bacterium *Escherichia coli* and in tobacco plants (*Nicotiana tabacum*). This was done to determine whether functional enzyme expression was feasible in these organisms. In extracts of recombinant *E. coli* cells, five of the proteins were detected by western blot analysis, but exocellulases E3 and E6 were undetectable. In the plant-based expression system we were able to detect all six cellulases but not the β-glucosidase even though activity was detectable. When *E. coli* was used as the expression system, endocellulase E2 was active, while endocellulases E1 and E5 showed only residual activity. The remaining cellulases appeared completely inactive against the model substrates azo-carboxymethyl-cellulose (Azo-CMC) and 4-methylumbelliferyl-cellobioside (4-MUC). Only the β-glucosidase showed high activity against 4-MUC. In contrast, all the plant-derived enzymes were active against the respective model substrates. Our data indicate that some enzymes of bacterial origin are more active and more efficiently expressed in plants than in a bacterial host.

## Introduction

Energy security currently depends on fossil fuels, and the global challenges caused by our reliance on such non-renewable resources were highlighted when the oil price peaked in 2008. This emphasized the importance of research into renewable alternatives (Hamilton, 2009). Plant biomass provides an abundant and renewable source of feedstock for energy production which could be used to tackle these challenges and achieve a more secure energy supply (Yuan et al., 2008). Plants such as sugar cane and maize can be used to provide energy but they are also used as food and feed crops, so this creates conflict over the use of resources (Senauer, 2008). In contrast, the use of cellulose as a source of energy does not create such conflicts because lignocellulosic biomass is not used as food or feed. Cellulose is the most abundant biopolymer on earth (Goldstein, 1981; Lutzen et al., 1983) and cellulose-rich perennial grasses such as miscanthus or switchgrass can be grown on marginal land. This would help to avoid potential land use conflicts while providing an alternative renewable source of biomass for biofuel production.

Cellulose is recalcitrant to degradation and its use as a raw material for sugar production is therefore more challenging than the use of starch derived from maize (Jordan et al., 2012). A series of processes is required to separate the cellulose from other cell wall components, and enzyme mixtures are needed to achieve efficient polymer degradation. At least three classes of enzymes, endocellulases, exocellulases, and β-glucosidases, are required for efficient cellulose hydrolysis. The performance of these enzymes can be enhanced by additional enzymes such as accessory proteins, e.g., swollenin (Jäger et al., 2011; Jordan et al., 2012). The production costs for these enzymes must be reduced significantly to achieve economical lignocellulose valorization because they can contribute ∼40% of the overall process costs (Sainz, 2009).

Several strategies have been developed to reduce the cost of cellulases, including the production of recombinant enzymes in bacteria, yeast and plants (Garvey et al., 2013). Plants can be used as natural bioreactors to produce cellulases. This approach avoids the need for expensive synthetic growth media and allows inexpensive scale up by simply increasing the amount of land used for cultivation. For example, *Acidothermus cellulolyticus* endocellulase E1 has been expressed successfully in tobacco, alfalfa, rice, maize, and potato. These experiments indicated that cellulases can be expressed in plants and demonstrated that enzyme localization affects the yield (Ziegelhoffer et al., 2001; Egelkrout et al., 2012). Other reports describe the expression of cellulases from fungi such as *Trichoderma reesei*, bacteria such as *T. fusca* and the hyperthermophilic archeon *Sulfolobus solfataricus* (Jiang et al., 2011; Klose et al., 2012, 2013). Cellulases and accessory enzymes have also been expressed in tobacco plastids to produce the components of efficient cellulase cocktails (Verma et al., 2010). Some researchers claim expression levels of up to 40% of total soluble protein (TSP) for specific enzymes produced in the plastid system (Petersen and Bock, 2011).

The soil bacterium *T. fusca* is a well-studied organism and much research has been performed on its cellulolytic system (Gomez del Pulgar and Saadeddin, 2014). Although there have been approaches to use *T. fusca* enzymes in cellulosomes (Moraïs et al., 2010, 2012), there are no reports on the industrial use of the bacterium itself.

*Thermobifida fusca* cellulase genes have predominantly been used for plastid transformation rather than nuclear transformation. Here, we investigated the transient expression of *T. fusca* cellulolytic enzymes in the ER of tobacco cells to determine the feasibility of this approach. We compared the expression of six cellulases and one β-glucosidase in tobacco and *E. coli* to study the impact of eukaryotic and prokaryotic hosts on enzyme expression and activity. Our results showed that although the enzymes were derived from a bacterium, they were expressed more efficiently and with higher activity when targeted to the ER of plant cells.

## Materials and Methods

### PCR Amplification of Target Genes and Vector Construction

Seven genes encoding six secreted *T. fusca* cellulases and one β-glucosidase were amplified from *T. fusca* genomic DNA, i.e., endocellulases E1 (AAC06387, EMBL-CDS) and E2 (celB, AAC06388, EMBL-CDS), exocellulase E3 (cel6B, AAA62211, EMBL-CDS), the processive endocellulase E4 (celD, AAB42155, EMBL-CDS), endocellulase E5 (celE, AAZ54939.1, EMBL-CDS), exocellulase E6 (celF, AAD39947, EMBL-CDS) and β-glucosidase BglC (AAF37730, EMBL-CDS). The primers amplified a sequence corresponding to the mature region of each protein excluding the native signal peptide. Furthermore, they introduced NcoI or PciI restriction sites at the 5′ end and NotI sites at the 3′ end to generate the following seven products: PciI-BglC-NotI, NcoI-E1-NotI, NcoI-E2-NotI, PciI-E3-NotI, PciI-E4-NotI, NcoI-E5-NotI, and NcoI-E6-NotI. These were digested with the appropriate enzymes and ligated into the similarly treated pTRAkc-ERH vector (Maclean et al., 2007) to generate the transient expression vectors and add a His_6_ tag at C-terminus of each product.

The vector pJK was based on pRB95 (GenBank: AJ312393.1) (Ruf et al., 2001) which was generously provided by Prof. Ralph Bock. The plasmid was digested with SacII and ClaI for ligation with an expression cassette. This was amplified from pPAC-dsRed, based on pFaaDAII (Koop et al., 1996), using primers designed to add 5′ SacII and 3′ ClaI restriction sites. After digestion with the appropriate enzymes, the cassette was ligated into vector pRB95 to generate the final construct pJK01. This vector provides a shuttle system which can be used for expression in *E. coli* as well as for chloroplast transformation.

The E1, E2, E5, and E6 genes were transferred to vector pTRAkc-TP using NcoI and NotI. These intermediate vectors were digested with NcoI and XbaI to isolate the genes including the C-terminal His_6_ tag sequences. The products were transferred to vector pJK01 to generate the final constructs pJK-E1, pJK-E2, pJK-E5, and pJK-E6. The BglC, E3 and E4 genes were transferred to pTRAkc-TP using PciI and NotI. The sequences in the intermediate vectors were amplified using gene-specific forward primers combined with the Cel universal reverse primer to yield the BglC, E3 and E4 products including C-terminal His_6_ tag sequences. These products were digested with PciI and XbaI and transferred to the similarly treated pJK01 vectors to generate the final constructs pJK-BglC, pJK-E3 and pJK-E4.

All the vectors listed above were sequenced to confirm the correct sequences before the transformation of *E. coli* DH5 α cells. All primers discussed above are listed in **Table 1**.

**Table 1:** Primers used for gene amplification.

Prime name	Sequence 5′→3′
Tfu BglC fw	ATACATGTTGACCTCGCAATCGACGACTCC
Tfu BglC rv	AATGCGGCCGCTTCCTGTCCGAAGATTCC
Tfu E1 fw	AGACCATGGACGAAGTCAACCAGATTCGCAAC
Tfu E1 rv	ATAAGCGGCCGCGCCGATGGAGCAGAC
Tfu E2 fw	AGACCATGGCCAATGATTCTCCGTTCTACGTCAACCC
Tfu E2 rv	ATAGCGGCCGCGCTGGCGGCGCAGGTAAG
Tfu E3 fw	TATTACATGTTAGCCGGCTGCTCGGTGG
Tfu E3 rv	ATATGCGGCCGCCAGAGGCGGGTAGGCG
Tfu E4 fw	ATAACATGTTAGAACCGGCGTTCAACTACGCCG
Tfu E4 rv	ATAGCGGCCGCGGCGAGGGCGCAG
Tfu E5 fw	ATACCATGGGTCTCACCGCCACAGTCACC
Tfu E5 rv	ATAAGCGGCCGCGGACTGGAGCTTGCTC
Tfu E6 fw	AATCCATGGCGGCCGTCGCCTGCTC
Tfu E6 rv	ATAATGCGGCCGCGGGAGCTCCGGCCCC
Cel rv	CTGACTCTAGAGGATCCGAGCTCGAGC

### Cultivation of Bacteria and Protein Extraction

Transformed bacteria were grown overnight in 10 ml LB medium (100 μg/ml Ampicillin) as preculture and main culture was inoculated with OD_600_ of 0.1 in 100 ml LB medium (100 μg/ml Ampicillin) and grown over night at 37°C. Cells were harvested by centrifugation (10 min, 5000 × *g*, 4°C) after measuring the final OD_600_ of the cultures. Cell pellets were resuspended in 2 mL PBS, increasing the concentration 50 fold, and lysed by sonication. Cell debris were separated from supernatant by centrifugation (20 min, 17,500 × *g*, 4°C).

Total soluble protein content of the extracts was determined using the Bradford method (Bradford, 1976). Equal amounts of protein were used for further experiments. Cellulase expression was confirmed by SDS-PAGE for 50 min at 200 V followed by western blotting as previously described (Laemmli, 1970; Burnette, 1981). The recombinant proteins were detected with a monoclonal mouse anti-polyhistidine antibody (Qiagen) diluted 1:5000 as primary antibody. A secondary monoclonal goat anti-mouse antibody conjugated with alkaline phosphatase (Dianova) also diluted 1:5000, with nitroblue tetrazolium and 5-bromo-4-chloro-3-indolyl phosphate (Roth) was used for visualization.

The cell pellet was resuspended subsequently in an equal volume of PBS and used for another SDS-PAGE and western blotting to detect insoluble protein.

### Transient Protein Expression Analysis in Plants

*Agrobacterium tumefaciens* strain GV3101::pMP90RK (Koncz and Schell, 1986) was transformed with the expression constructs described above according to established procedures (Shen and Forde, 1989). The resulting clones were incubated for ca. 36 h in YEB medium (kanamycin (50 μg/ml), rifampicin (50 μg/ml) and carbenicillin (100 μg/ml)) at 26°C. From these cultures, 200 μl of the cultures were used to inoculate 20 ml of YEB medium. Bacteria were grown again for ca. 36 h at 26°C. Then a preinduction was performed by addition of 2 μL acetosyringon (200 μM final concentration), 100 μL 40% (w/v) glucose solution, and 200 μL of 1 M MES buffer pH 5.6. The culture was further grown over night and the OD_600_ was determined. The cultures were diluted up to 15 ml with deionized water. Then 15 ml of 2x infiltration buffer (100 g/L sucrose, 3.6 g/L glucose, 8.6 g/L Murashige and Skoog basal MS salts, pH 5.6) were added to obtain a final OD_600_ of 1. These infiltration solutions were used to infiltrate a single leaf of four independent 6 weeks old *Nicotiana tabacum* SR1 plants (Kapila et al., 1997; Garvey et al., 2014). Each leaf was of different age. The plants were cultivated for four further days at 25°C. For each construct, 0.5 g of infiltrated leaf material from each leaf were used for protein extraction. The samples were ground in 1 ml PBS and centrifuged (20 min, 21000 × *g*, 4°C). After this, 250 μl of leaf extract from each leaf corresponding to a construct were unified to obtain a more evenly distributed protein extract.

Total soluble protein content of the extracts was determined via Bradford assay, as described above and equal amounts of TSP were used in subsequent experiments. Cellulase expression was confirmed by SDS-PAGE followed by western blotting, as described above for the *E. coli* derived samples.

*N*-glycosylation sites were predicted using NetNGlyc 1.0 server software^1^ based on the mature amino acid sequence of the proteins predicted from the DNA sequence data.

### Enzyme Activity Assays

Enzyme activity was determined using Azo-CMC (Megazyme) and 4-MUC (Sigma) as model substrates to measure the endocellulase and exocellulase activities of the enzymes, respectively (Ziegelhoffer et al., 2001; Jørgensen et al., 2004). For the Azo-CMC assay, 52 μg of the bacterial protein extract and 5.625 μg of the leaf extract were added to a 500 μL 0.5% (w/v) Azo-CMC in 50 mM sodium acetate (pH 5.5). Hydrolysis was carried out at 50°C for 30 min then stopped by adding 1.25 mL precipitation buffer. After centrifugation (10 min, 1000 × *g*, room temperature), the absorbance of the supernatant at 590 nm was measured photometrically and the enzyme activities were calculated against a calibration curve established using defined amounts of Onozuka Cellulase mix (Serva).

For the 4-MUC assay, 5.625 μg of raw plant extract and 52 μg of raw bacterial extract were mixed with 100 μL 50 mM sodium acetate buffer (pH 5.5) containing 0.5 mM 4-MUC and incubated at 50°C for 2 h. The assay was stopped by adding 100 μL 0.15 M glycine (pH 10) and fluorescence was measured at 465 nm (excitation, 360 nm) against a calibration curve. For both assays, enzymatic activity was compared to empty vector controls (pRB95 for *E. coli* and pTRAkc for tobacco).

## Results

### Cloning and Sequencing Analyses

**FIGURE 1:**
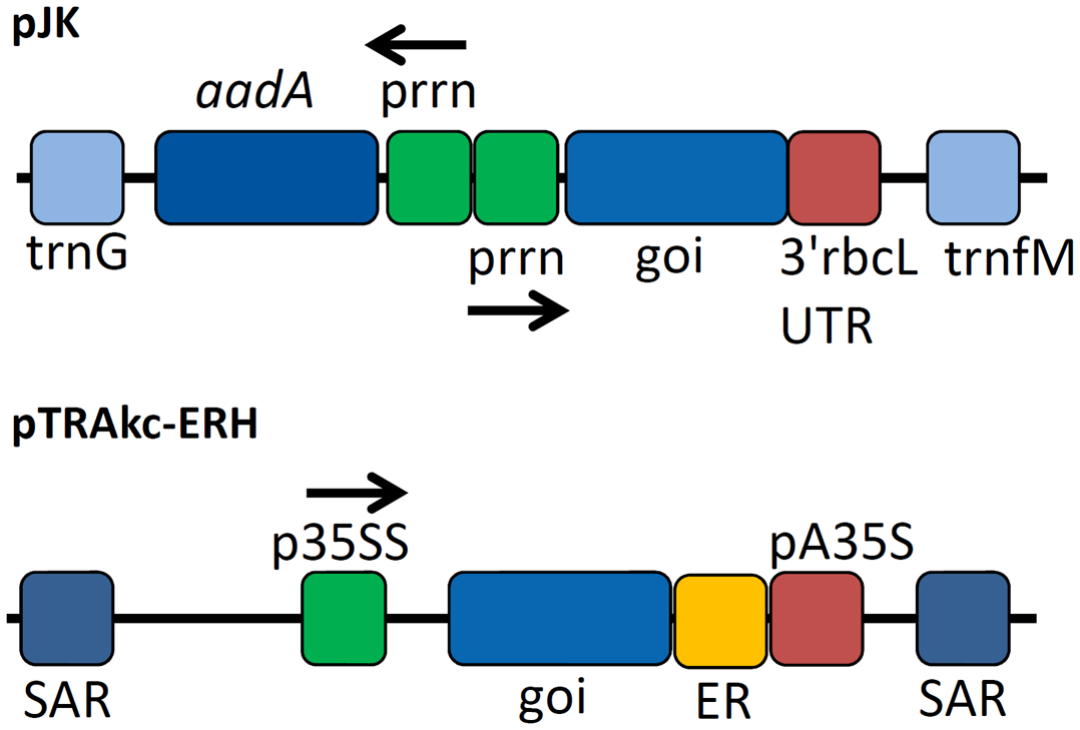
**Expression cassettes used for cellulase expression**. Expression cassettes based on pJK contained sequences derived from the *Nicotiana tabacum* chloroplast genes for tRNA-Gly (*trn*G) and tRNA-Met (*trn*M) used as homologous regions for recombination into the chloroplast genome of *N. tabacum* after particle bombardment as well as copies of the plastid ribosomal promoter (p*rrn*) for expression of selectable marker gene aminoglycoside 3′-adenylyltransferase (*aad*A) and the gene of interest (goi) using 3′rbcL (ribulose bisphosphate carboxylase large subunit from *Chlamydomonas reinhardtii*) UTR as 3′ untranslated region. The cassette for the transient expression experiments contained scaffold attachment regions (SARs) and the p35SS promoter controlling transgene expression with an additional retention signal for localization in the endoplasmic reticulum (ER) and pA35S as polyadenylation signal.

Seven target genes were successfully amplified by PCR and transferred to the target vectors pJK and pTRAkc-ERH for bacterial and plant transformation, respectively (**Figure 1**). The vectors included a polyhistidine coding sequence that was added to the 3′-end of each coding region to generate a C-terminal His_6_ tag. Sequencing the new plasmids revealed some differences compared to previously published sequences (Lao et al., 1991; Jung et al., 1993; Zhang et al., 1995; Irwin et al., 2000; Lykidis et al., 2007). The endocellulase E2 gene featured a silent mutation (30V) whereas the endocellulase E1 gene included a nucleotide exchange causing an amino acid substitution (174R→G) and also a deletion that removed residue 944G. The genes for E3 and E6 also contained mutations that caused amino acid substitutions (437N→D for E3 and 194I→T for E6). The remaining two cellulase genes and the sequence for the β-glucosidase (BglC) were identical to published data. Because the detected mutations were present in multiple clones for each gene, we assumed they represented naturally occurring alleles rather than PCR artifacts.

### Protein Expression in *N. tabacum* and in *E. coli*

**FIGURE 2:**
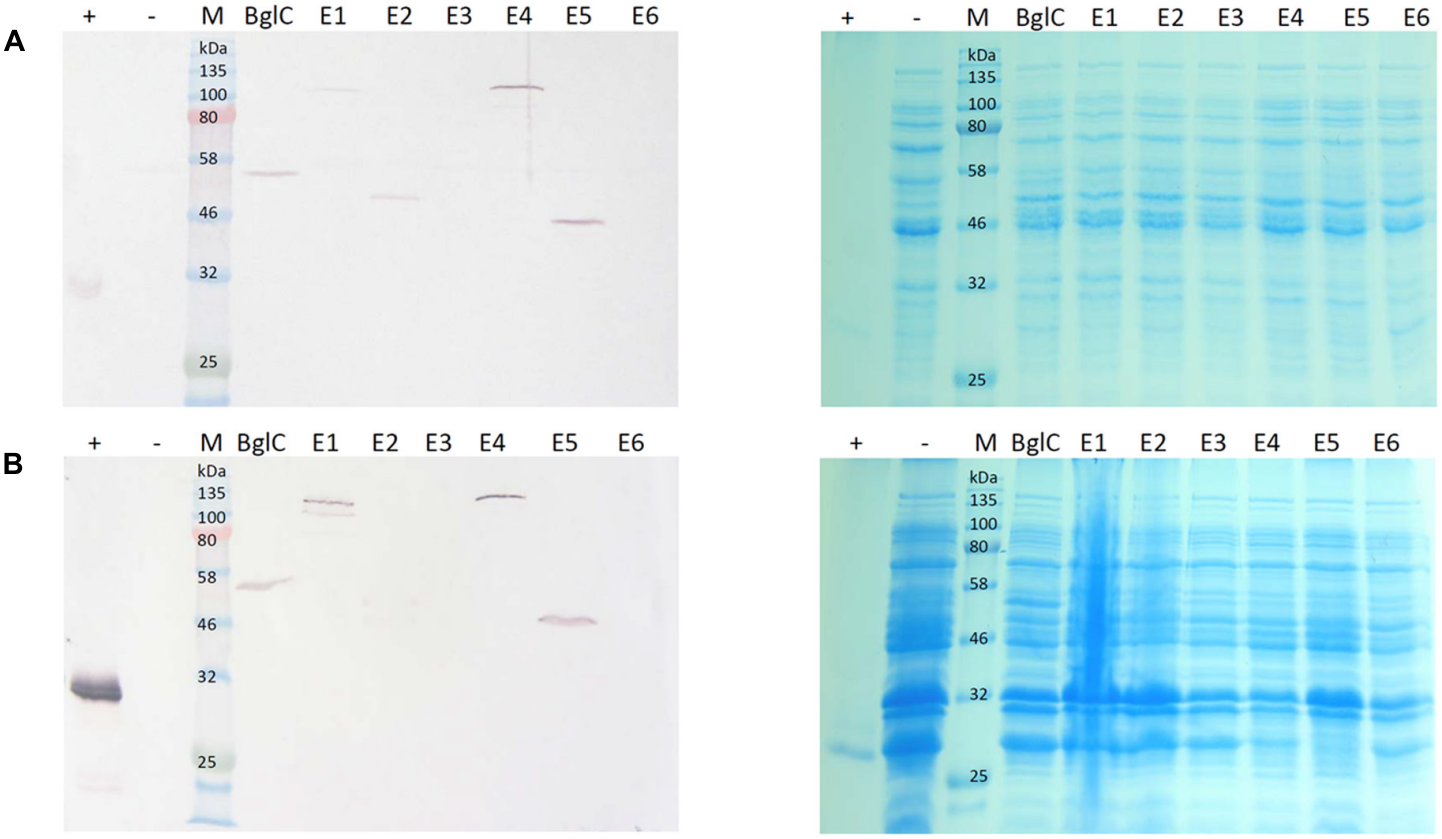
**Western blot and Coomassie stained gel of *Escherichia coli* cell extracts and cell pellet fractions**. Proteins were detected with monoclonal anti-polyhistidine antibodies. His-tagged mCherry was used as a positive control (+) and a cell extract from *E. coli* transformed with an empty vector (-) was used as a negative control. Lanes for BglC and E1–E6 contain cell extracts from cultures transformed with the corresponding pJK vectors for cellulase expression. Per lane, 5 μg total soluble protein (TSP) **(A)** or 15 μg TSP **(B)** were loaded. **(A)** Depicts a blot of the cell lysate with the corresponding Coomassie stained gel. **(B)** Depicts a blot of the cell pellet fraction with the corresponding Coomassie stained gel.

Samples were harvested from *E. coli* cultures and *N. tabacum* leaves and the resulting extracts were used directly for western blotting and activity assays. To detect possible negative effects on *E. coli* growth behavior related to cellulase expression, OD_600_ at the end of the cultivation time was measured and compared to the empty vector control. **Table 2** shows the average OD_600_ measured from four independent cultivations. This showed no clear negative effect of the cellulase expression on the final cell density. The bacterial extracts yielded bands in the western blot indicating the successful expression of all genes except those encoding exocellulases E3 and E6 (**Figure 2A**). The mobility of each band was consistent with the theoretical mass of the corresponding protein (**Table 3**), showing the successful expression of full-length proteins. Furthermore, each construct produced a single band on the blot suggesting the absence of protein degradation products. The blot for the cell pellet fraction (**Figure 2B**) showed bands only for BglC, E1, E4, and E5 indicating that a part of the protein remains insoluble. An additional band was observed for E1 indicating a slight degradation or a potential isoform of the enzyme when present in the insoluble fraction. For the exocellulases E3 and E6 as well as the endocellulase E2 there were no bands observed.

**Table 2:** Final OD_600_ measured for *Escherichia coli* expression cultures.

	OD_600_
pRB95	5.33 ± 0.16
BglC	4.90 ± 0.26
E1	4.84 ± 0.73
E2	5.41 ± 0.21
E3	5.15 ± 0.33
E4	5.20 ± 0.19
E5	5.17 ± 0.49
E6	5.23 ± 0.31

**Table 3:** Theoretical masses of expressed enzymes.

BglC	54.6 kDa
E1	102.5 kDa
E2	44.2 kDa
E3	60.9 kDa
E4	91.7 kDa
E5	47.5 kDa
E6	104.9 kDa

**FIGURE 3:**
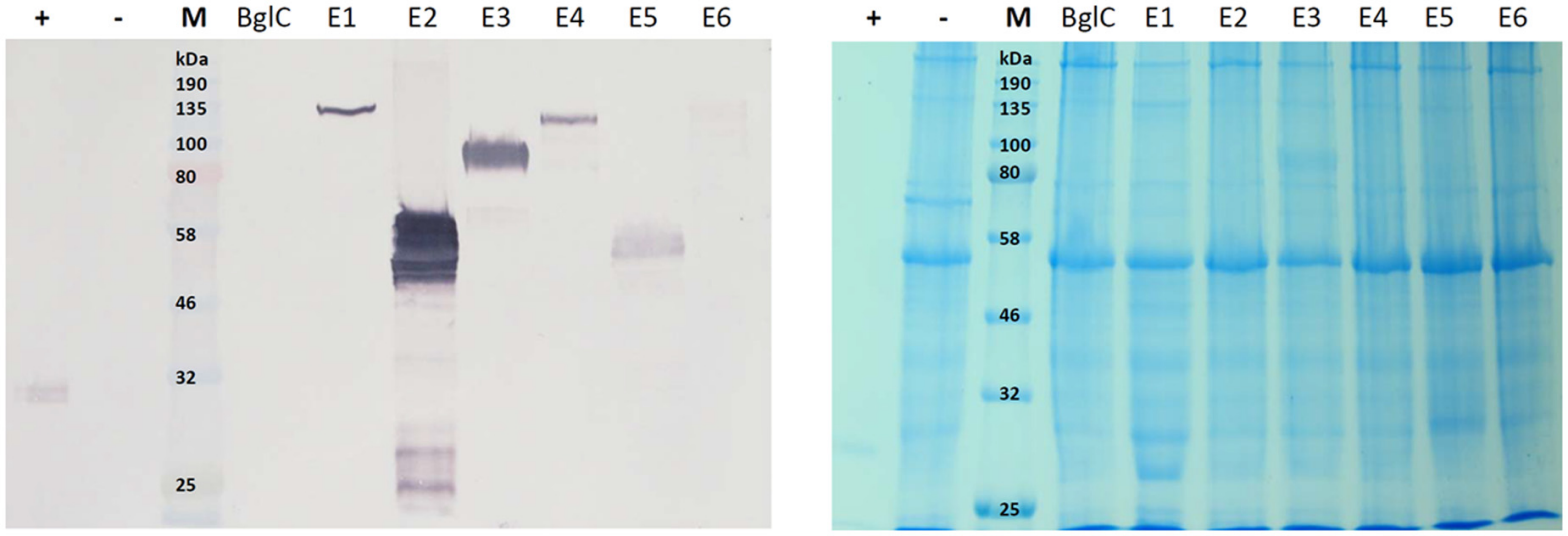
**Western blot and Coomassie stained gel of *N. tabacum* leaf extracts**. Proteins were detected with monoclonal anti-polyhistidine antibodies. His-tagged mCherry was used as a positive control (+) and leaf extract of a plant infiltrated with the empty vector pTRAkc (-) was used as negative control. Lanes for BglC and E1–E6 contain leaf extracts of plants infiltrated with the respective pTRAkc-ERH clones for cellulase expression. Each sample represents 5.625 μg of TSP (total soluble protein).

The leaf extracts (**Figure 3**) yielded bands for all six cellulases, but no band was visible for β-glucosidase BglC. We observed a single ∼135 kDa band for endocellulase E1 and multiple bands in the range of 48–65 kDa for endocellulase E2, as well as weaker bands (∼28 – 24 kDa) potentially showing evidence of protein degradation. For exocellulase E3 and the processive endocellulase E4, bands were visible at ∼90 kDa and ∼110 kDa, respectively. Weaker E4 bands (∼100 kDa and ca. 80 kDa) represented additional forms of the protein, whereas the additional Band received for E3 (∼65 kDa) might represent a non-glycosylated form of the exocellulase. E5 was represented by a strong band at ∼55 kDa and several weak smaller bands potentially representing degradation fragments. Exocellulase E6 was represented by a ∼120 kDa band and a weaker band at about 100 kDa. The high molecular mass of the main enzyme bands suggested that (partial) *N*-glycosylation had occurred in the ER, although none of these proteins are glycosylated in their natural host *T. fusca*. Subsequently, *N*-glycosylation analysis was performed, using the NetNGlyc 1.0 Server software to predict glycan acceptor sites. As shown in **Table 4**, all of the enzymes contain 1–8 likely glycosylation sites, suggesting that glycosylated forms are probable to be synthesized in plants. This correlates well with the higher molecular masses of the modified protein variants detected by western blot.

**Table 4:** Predicted *N*-glycosylation sites for *Thermobifida fusca* enzymes; +, ++, and +++ show an increasing likelihood of glycosylation.

	+	++	+ + +
BglC	1	0	0
E1	3	2	1
E2	3	2	1
E3	3	2	3
E4	2	0	0
E5	1	2	0
E6	2	1	1

*Sites unlikely to be glycosylated were omitted*.

### Activity Assays on Model Substrates 4-MUC and Azo-CMC

**FIGURE 4:**
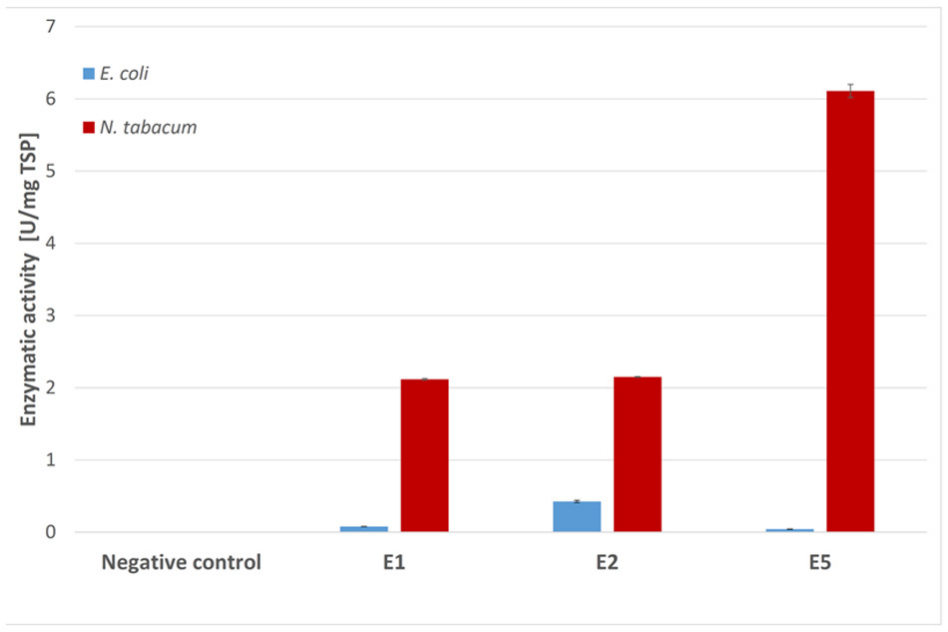
**Azo-CMC assay for endocellulase activity**. Each 500 μl reaction was carried out for 30 min at 50°C in 50 mM sodium acetate buffer (pH 5.5) containing 0.5% Azo-CMC (w/v) and values were calculated for 1 mg TSP of cell or leaf extract for better comparison. The reaction was stopped by addition of 1.25 ml precipitation solution. After centrifugation (10 min, 1000 × *g*, room temperature), the absorbance of the supernatant at 590 nm was measured photometrically. All measurements were performed in triplicate. Error bars show standard deviation.

Endocellulases E1, E2, and E5 were tested for endocellulase activity using the model substrate azo-carboxymethyl-cellulose (Azo-CMC). The results for each expression system were adjusted to 1 mg of TSP and were compared in **Figure 4**. Although E2 clearly showed activity against the test substrate when produced in *E. coli*, we observed almost no detectable activity for E1 and E5 in this expression system. Plant extracts containing the three endocellulases were active against Azo-CMC, and all extracts were more active than their recombinant bacterial counterparts when adjusted to equal TSP content.

**FIGURE 5:**
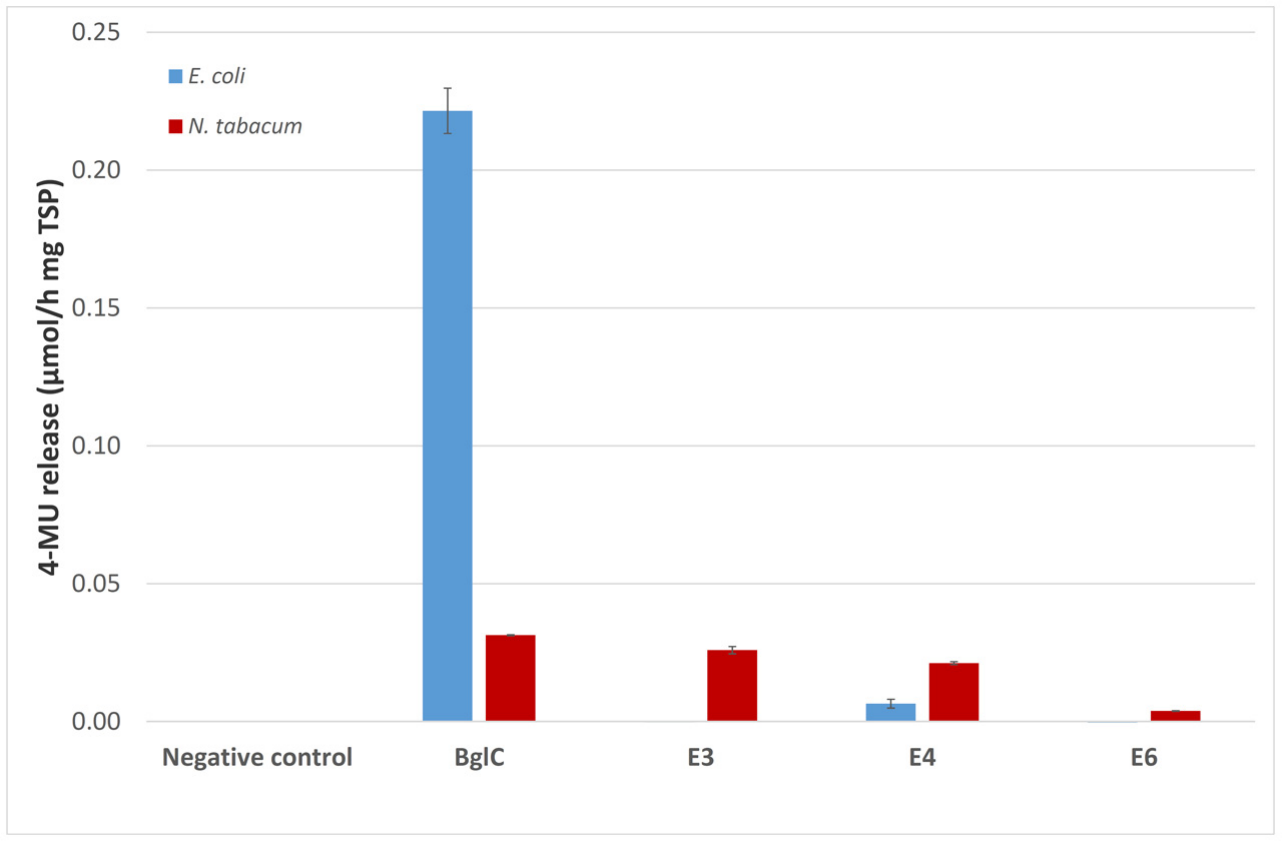
**The 4-MUC assay for enzyme activity**. Each 100 μl reaction was carried out for 2 h at 50°C in 50 mM sodium acetate buffer (pH 5.5) containing 1 mM 4-MUC and values were calculated for 1 mg TSP of cell or leaf extract for better comparison. The assay was stopped by adding 100 μL 0.15 M glycine (pH 10) and fluorescence was measured at 465 nm (excitation, 360 nm) against a calibration curve. All measurements were performed in triplicate. Error bars show standard deviation.

The activity of the remaining enzymes was tested against 4-MUC (**Figure 5**). The bacterial extracts showed no detectable activity for the remaining cellulases compared to the empty vector control (pRB95). In contrast, the activity of BglC exceeded the detection limit of the assay indicating that the enzyme was highly active when produced in *E. coli*. All the cellulases appeared to be active compared to the negative control (empty pTRAkc vector) when expressed in tobacco, but the activity of plant-derived BglC was significantly lower than the same enzyme produced in *E. coli* when adjusted to similar amounts of TSP.

## Discussion

### Comparison of ER Targeted Expression of *T. fusca* Cellulases in *N. tabacum* to Cytosolic Expression in *E. coli*

We evaluated the potential industrial use of *T. fusca* cellulases by expressing the individual recombinant enzymes in *E. coli* and *N. tabacum* using vectors based on pRB95 and pTRAkc-ERH. The pRB95 based vectors were used for bacterial expression as the utilized constitutive plastid ribosomal RNA promoter (p*rrn*) promoter is known to be recognized in *E. coli* (Drechsel and Bock, 2011). Most of the proteins were expressed successfully in *E. coli* but we were unable to detect the exocellulases. This indicated a product-specific effect rather than a general problem with the bacterial expression system. Furthermore, all six cellulases were expressed successfully in tobacco plants, confirming that the absence of exocellulases in the *E. coli* extracts did not reflect a problem with the amplified gene sequence. An additional blot of the bacterial cell pellet fraction revealed bands for enzymes which were also present in the soluble fraction but no evidence was found for the presence of the exocellulases. This indicates that the exocellulases were rather degraded than accumulated in inclusion bodies. On the other hand, exocellulases E3 was expressed in *E. coli* previously (Zhang et al., 2000; Jeoh et al., 2002). In these cases, the expression of the gene was performed on a larger scale (6 L). This fact might indicate that the expression was also rather low in these cases.

Both exocellulases contain four cysteine residues. Disulfide bond formation has been confirmed for E3 (Rouvinen et al., 1990) while E6 lacked free SH groups when expressed in *Streptomyces lividans* (Irwin et al., 2000). Therefore, the impairment of disulfide bond formation in the *E. coli* cytosol is a reasonable explanation for the observed lack of exocellulase expression in this system (de Marco, 2009). The plant ER environment promotes disulfide bond formation (Onda et al., 2009; Lombardi et al., 2012) supporting the hypothesis that exocellulase accumulation in *E. coli* is prevented by the inability to form disulfide bonds efficiently. This might lead to the degradation of unfolded proteins. Thus, the ER compartment was chosen for cellulase expression in tobacco to enable proper protein folding and to prevent degradation by proteases as documented for alternative compartments in other studies (Dai et al., 2000; Ziegelhoffer et al., 2001).

The initial expression of *T. fusca* cellulases in *E. coli* was challenging, and *Streptomyces lividans* was used as an alternative host (Ghangas and Wilson, 1987). More recent studies have achieved the expression of certain *T. fusca* cellulases in *E. coli* (Vuong and Wilson, 2009; Li et al., 2010) but more detailed data on the yield and activity are only available for endocellulase E5 (Yan et al., 2013). Based on our western blot data, the p*rrn* promoter used in our expression construct is suitable for the efficient expression of *T. fusca* endocellulases in *E. coli*.

High yields of all six cellulases were achieved in tobacco, but this was not the case for the β-glucosidase BglC. Notably, this enzyme is the only protein in the set that is not secreted naturally by its host, and this may explain its weak expression in the ER of tobacco cells. Cellulase E1 yielded a strong single band in western blots of plant extracts. The remaining cellulases yielded additional weak bands with lower molecular weights than the main band, suggesting they could be degradation products or less glycosylated versions of the enzymes. The expression of full-size *T. fusca* enzymes in tobacco is therefore feasible. In contrast, only the catalytic domain of *Acidothermus cellulolyticus* E1 was present in maize and tobacco extracts reflecting extensive degradation (Ziegelhoffer et al., 2001; Hood et al., 2012). The successful expression of *T. fusca* cellulases in the ER suggests that this compartment promotes higher yields, higher enzyme activities and higher protein stability compared to cytosolic expression (Ziegelhoffer et al., 1999). The altered mobility of the enzymes during SDS-PAGE suggested the proteins may be glycosylated, which is consistent with the multiple bands representing E2 and the *in silico* prediction of *N*-glycosylation sites indicating 1–8 likely *N*-glycosylation sites per enzyme. This hypothesis is supported further by the results of the bacterial expression where the enzymes showed the expected molecular weights. As glycosylation can influence the activity and stability of recombinant proteins (Beckham et al., 2012), its impact on the enzymes included in our study will be investigated in future experiments.

### Activity Assays on Model Substrates for Enzymes Expressed in Tobacco and *E. coli*

Our enzyme activity assays showed that only BglC and the endocellulases E1, E2, and E5 were active when expressed in *E. coli*, although a band representing E4 was observed on western blots confirming the presence of the protein in the cell extracts. This enzyme was produced as full-length protein, and should therefore show activity against model substrate, which was clearly not the case. The inactivity of E4 may be a consequence of incorrect protein folding caused by the inefficient formation of disulfide bonds in *E. coli*, as also proposed to explain the absence of two exocellulases in this species. Furthermore this might be the reason for the low activities observed for E1 and E5 observed in the Azo-CMC assay. The plant-derived versions of all the cellulases were active, indicating that the lack of activity does not reflect any sequence-dependent properties and further supporting the hypothesis that the *E. coli* cytoplasm provides an inadequate environment for these enzymes. These data suggest that cellulase activity in the natural host *T. fusca* therefore depends on the efficient formation of disulfide bonds as it was shown for the *T. fusca* E2 by McGinnis and Wilson (1993).

Plant extracts showed activity for all seven enzymes (including BglC, which was not detected by western blot) thus correlating with the strong BglC activity detected in *E. coli*. The western blot and activity assay data for the plant-derived cellulases indicated that the higher protein masses potentially caused by glycosylation did not influence the activity of any of the enzymes, suggesting that tobacco could be used to produce active *T. fusca* cellulases without modifying the glycosylation pathway. It is not yet clear whether nuclear transformation will equal the efficiency of plastid transformation for the expression of *T. fusca* enzymes (Yu et al., 2007; Gray et al., 2009, 2011; Petersen and Bock, 2011) and these approaches will be compared in future experiments.

Our data confirm the successful expression of active bacterial cellulases in the tobacco ER without any of the major degradation issues reported for cellulases derived from other bacterial sources. We were able to produce a full set of cellulolytic enzymes secreted by *T. fusca* in plants. This work comprises a good foundation for more detailed studies in the future to facilitate the inexpensive production of functional heterologous cellulolytic complexes for the efficient production of biofuels from lignocellulosic biomass.

## Author Contributions

JK designed and carried out the experiments, analyzed results and wrote the manuscript. RF and UC coordinated the study and reviewed the manuscript. All authors read and approved the final manuscript.

## Conflict of Interest Statement

The authors declare that the research was conducted in the absence of any commercial or financial relationships that could be construed as a potential conflict of interest.

## Footnotes

1 http://www.cbs.dtu.dk/services/NetNGlyc/

## ABBREVIATIONS

4-MUC: 4-Methylumbelliferyl β-D-cellobioside
Azo-CMC: Azo-carboxymethyl cellulose
ER: endoplasmic reticulum

## References

[B1] Beckham G. T. Dai Z. Matthews J. F. Momany M. Payne C. M. Adney W. S. (2012). Harnessing glycosylation to improve cellulase activity. *Curr. Opin. Biotechnol.* 23 338–345. 10.1016/j.copbio.2011.11.030 22186222

[B2] Bradford M. M. (1976). A rapid and sensitive method for the quantitation of microgram quantities of protein utilizing the principle of protein-dye binding. *Anal. Biochem.* 72 248–254. 10.1016/0003-2697(76)90527-3 942051

[B3] Burnette W. N. (1981). “Western Blotting”: electrophoretic transfer of proteins from sodium dodecyl sulfate-polyacrylamide gels to unmodified nitrocellulose and radiographic detection with antibody and radioiodinated protein A. *Anal. Biochem.* 112 195–203. 10.1016/0003-2697(81)90281-5 6266278

[B4] Dai Z. Hooker B. S. Anderson D. B. Thomas S. R. (2000). Improved plant-based production of E1 endoglucanase using potato: expression optimization and tissue targeting. *Mol. Breed.* 6 277–285. 10.1023/A:1009653011948

[B5] de Marco A. (2009). Strategies for successful recombinant expression of disulfide bond-dependent proteins in *Escherichia coli*. *Microbial Cell Fact.* 8:26 10.1186/1475-2859-8-26 PMC2689190 19442264

[B6] Drechsel O. Bock R. (2011). Selection of shine-dalgarno sequences in plastids. *Nucleic Acids Res.* 39 1427–1438. 10.1093/nar/gkq978 20965967 PMC3045613

[B7] Egelkrout E. Mcgaughey K. Keener T. Ferleman A. Woodard S. Devaiah S. (2012). Enhanced expression levels of cellulase enzymes using multiple transcription units. *BioEnergy Res.* 6 699–710. 10.1007/s12155-012-9288-x

[B8] Garvey M. Klinger J. Klose H. Fischer R. Commandeur U. (2014). Expression of recombinant cellulase cel5a from trichoderma reesei in tobacco plants. *J. Vis. Exp.* 13:e51711 10.3791/51711 PMC4189538 24962636

[B9] Garvey M. Klose H. Fischer R. Lambertz C. Commandeur U. (2013). Cellulases for biomass degradation: comparing recombinant cellulase expression platforms. *Trends Biotechnol.* 31 581–593. 10.1016/j.tibtech.2013.06.006 23910542

[B10] Ghangas G. S. Wilson D. B. (1987). Expression of a *Thermomonospora fusca* Cellulase Gene in *Streptomyces* lividans and *Bacillus subtilis*. *Appl. Environ. Microbiol.* 53 1470–1475. 16347376 10.1128/aem.53.7.1470-1475.1987 PMC203894

[B11] Goldstein I. S. (1981). *Organic Chemicals from Biomass.* Boca Raton, FL: CRC Press, Inc, 310.

[B12] Gomez del Pulgar E. M. Saadeddin A. (2014). The cellulolytic system of *Thermobifida fusca*. *Crit. Rev. Microbiol.* 40 236–247. 10.3109/1040841X.2013.776512 23537325

[B13] Gray B. N. Ahner B. A. Hanson M. R. (2009). High-level bacterial cellulase accumulation in chloroplast-transformed tobacco mediated by downstream box fusions. *Biotechnol. Bioeng.* 102 1045–1054. 10.1002/bit.22156 18973281

[B14] Gray B. Yang H. Ahner B. Hanson M. (2011). An efficient downstream box fusion allows high-level accumulation of active bacterial beta-glucosidase in tobacco chloroplasts. *Plant Mol. Biol.* 76 345–355. 10.1007/s11103-011-9743-7 21279422

[B15] Hamilton J. D. (2009). Causes and consequences of the oil shock of 2007-08. *Nat. Bur. Econ. Res. Work. Paper Ser.* 40 215–283.

[B16] Hood E. E. Devaiah S. P. Fake G. Egelkrout E. Teoh K. Requesens D. V. (2012). Manipulating corn germplasm to increase recombinant protein accumulation. *Plant Biotechnol. J.* 10 20–30. 10.1111/j.1467-7652.2011.00627.x 21627759

[B17] Irwin D. C. Zhang S. Wilson D. B. (2000). Cloning, expression and characterization of a Family 48 exocellulase, Cel48A, from *Thermobifida fusca*. *Eur. J. Biochem.* 267 4988–4997. 10.1046/j.1432-1327.2000.01546.x 10931180

[B18] Jäger G. Girfoglio M. Dollo F. Rinaldi R. Bongard H. Commandeur U. (2011). How recombinant swollenin from *Kluyveromyces lactis* affects cellulosic substrates and accelerates their hydrolysis. *Biotechnol. Biofuels* 4:33 10.1186/1754-6834-4-33 PMC3203333 21943248

[B19] Jeoh T. Wilson D. B. Walker L. P. (2002). Cooperative and competitive binding in synergistic mixtures of *Thermobifida fusca* cellulases Cel5A, Cel6B, and Cel9A. *Biotechnol. Prog.* 18 760–769. 10.1021/bp0200402 12153310

[B20] Jiang X.-R. Zhou X.-Y. Jiang W.-Y. Gao X.-R. Li W.-L. (2011). Expressions of thermostable bacterial cellulases in tobacco plant. *Biotechnol. Lett.* 33 1797–1803. 10.1007/s10529-011-0642-4 21618025

[B21] Jordan D. B. Bowman M. J. Braker J. D. Dien B. S. Hector R. E. Lee C. C. (2012). Plant cell walls to ethanol. *Biochem. J.* 442 241–252. 10.1042/BJ20111922 22329798

[B22] Jørgensen H. Mørkeberg A. Krogh K. B. R. Olsson L. (2004). Growth and enzyme production by three *Penicillium* species on monosaccharides. *J. Biotechnol.* 109 295–299. 10.1016/j.jbiotec.2003.12.011 15066767

[B23] Jung E. D. Lao G. Irwin D. Barr B. K. Benjamin A. Wilson D. B. (1993). DNA sequences and expression in *Streptomyces* lividans of an exoglucanase gene and an endoglucanase gene from *Thermomonospora fusca*. *Appl. Environ. Microbiol.* 59 3032–3043. 8215374 10.1128/aem.59.9.3032-3043.1993 PMC182403

[B24] Kapila J. De Rycke R. Van Montagu M. Angenon G. (1997). An *Agrobacterium*-mediated transient gene expression system for intact leaves. *Plant Sci.* 122 101–108. 10.1016/S0168-9452(96)04541-4

[B25] Klose H. Günl M. Usadel B. Fischer R. Commandeur U. (2013). Ethanol inducible expression of a mesophilic cellulase avoids adverse effects on plant development. *Biotechnol. Biofuels* 6:53 10.1186/1754-6834-6-53 PMC3643885 23587418

[B26] Klose H. Röder J. Girfoglio M. Fischer R. Commandeur U. (2012). Hyperthermophilic endoglucanase for in planta lignocellulose conversion. *Biotechnol. Biofuels* 5:63 10.1186/1754-6834-5-63 PMC3497586 22928996

[B27] Koncz C. Schell J. (1986). The promoter of TL-DNA gene 5 controls the tissue-specific expression of chimaeric genes carried by a novel type of *Agrobacterium* binary vector. *Mol. Gen. Genet.* 204 383–396. 10.1007/BF00331014

[B28] Koop H.-U. Steinmüller K. Wagner H. Rößler C. Eibl C. Sacher L. (1996). Integration of foreign sequences into the tobacco plastome via polyethylene glycol-mediated protoplast transformation. *Planta* 199 193–201. 10.1007/BF00196559 8680308

[B29] Laemmli U. K. (1970). Cleavage of structural proteins during the assembly of the head of bacteriophage T4. *Nature* 227 680–685. 10.1038/227680a0 5432063

[B30] Lao G. Ghangas G. S. Jung E. D. Wilson D. B. (1991). DNA sequences of three beta-1,4-endoglucanase genes from *Thermomonospora fusca*. *J. Bacteriol.* 173 3397–3407. 1904434 10.1128/jb.173.11.3397-3407.1991 PMC207951

[B31] Li Y. Irwin D. C. Wilson D. B. (2010). Increased crystalline cellulose activity via combinations of amino acid changes in the family 9 catalytic domain and family 3c cellulose binding module of *Thermobifida fusca* Cel9A. *Appl. Environ. Microbiol.* 76 2582–2588. 10.1128/AEM.02735-09 20173060 PMC2849196

[B32] Lombardi A. Marshall R. S. Castellazzi C. L. Ceriotti A. (2012). Redox regulation of glutenin subunit assembly in the plant endoplasmic reticulum. *Plant J.* 72 1015–1026. 10.1111/tpj.12020 22966775

[B33] Lutzen N. W. Nielsen M. H. Oxenboell K. M. Schulein M. Stentebjerg-Olesen B. (1983). Cellulases and their application in the conversion of lignocellulose to fermentable sugars. *Philos. Trans. R. Soc. Lon. B Biol. Sci.* 300 283–291. 10.1098/rstb.1983.0005

[B34] Lykidis A. Mavromatis K. Ivanova N. Anderson I. Land M. Dibartolo G. (2007). Genome sequence and analysis of the soil cellulolytic actinomycete *Thermobifida fusca* YX. *J. Bacteriol.* 189 2477–2486. 10.1128/JB.01899-06 17209016 PMC1899369

[B35] Maclean J. Koekemoer M. Olivier A. J. Stewart D. Hitzeroth I. I. Rademacher T. (2007). Optimization of human papillomavirus type 16 (HPV-16) L1 expression in plants: comparison of the suitability of different HPV-16 L1 gene variants and different cell-compartment localization. *J. Gen. Virol.* 88 1460–1469. 10.1099/vir.0.82718-0 17412974

[B36] McGinnis K. Wilson D. B. (1993). Disulfide arrangement and functional domains of. beta.-1, 4-endoglucanase E5 from *Thermomonospora fusca*. *Biochemistry* 32 8157–8161. 10.1021/bi00083a013 8347615

[B37] Moraïs S. Barak Y. Caspi J. Hadar Y. Lamed R. Shoham Y. (2010). Cellulase-xylanase synergy in designer cellulosomes for enhanced degradation of a complex cellulosic substrate. *mBio* 1:e00285-10. 10.1128/mBio.00285-10 PMC2999897 21157512

[B38] Moraïs S. Morag E. Barak Y. Goldman D. Hadar Y. Lamed R. (2012). Deconstruction of Lignocellulose into Soluble Sugars by Native and Designer Cellulosomes. *mBio* 3:e00508-12. 10.1128/mBio.00508-12 PMC3520109 23232718

[B39] Onda Y. Kumamaru T. Kawagoe Y. (2009). ER membrane-localized oxidoreductase Ero1 is required for disulfide bond formation in the rice endosperm. *Proc. Natl. Acad. Sci. U.S.A.* 106 14156–14161. 10.1073/pnas.0904429106 19666483 PMC2729036

[B40] Petersen K. Bock R. (2011). High-level expression of a suite of thermostable cell wall-degrading enzymes from the chloroplast genome. *Plant Mol. Biol.* 76 311–321. 10.1007/s11103-011-9742-8 21298465

[B41] Rouvinen J. Bergfors T. Teeri T. Knowles J. Jones T. (1990). Three-dimensional structure of cellobiohydrolase II from *Trichoderma reesei*. *Science* 249 380–386. 10.1126/science.249.4975.1359-e 2377893

[B42] Ruf S. Hermann M. Berger I. J. Carrer H. Bock R. (2001). Stable genetic transformation of tomato plastids and expression of a foreign protein in fruit. *Nat. Biotechnol.* 19 870–875. 10.1038/nbt0901-870 11533648

[B43] Sainz M. (2009). Commercial cellulosic ethanol: the role of plant-expressed enzymes. *In Vitro Cell. Dev. Biol. Plant* 45 314–329. 10.1007/s11627-009-9210-1

[B44] Senauer B. (2008). Food market effects of a global resource shift toward bioenergy. *Am. J. Agric. Econ.* 90 1226–1232. 10.1111/j.1467-8276.2008.01208.x

[B45] Shen W. J. Forde B. G. (1989). Efficient transformation of *Agrobacterium* spp. by high voltage electroporation. *Nucleic Acids Res.* 17:8385 10.1093/nar/17.20.8385 PMC334991 2682529

[B46] Verma D. Kanagaraj A. Jin S. Singh N. D. Kolattukudy P. E. Daniell H. (2010). Chloroplast-derived enzyme cocktails hydrolyse lignocellulosic biomass and release fermentable sugars. *Plant Biotechnol. J.* 8 332–350. 10.1111/j.1467-7652.2009.00486.x 20070870 PMC2854225

[B47] Vuong T. V. Wilson D. B. (2009). Processivity, synergism, and substrate specificity of *Thermobifida fusca* Cel6B. *Appl. Environ. Microbiol.* 75 6655–6661. 10.1128/AEM.01260-09 19734341 PMC2772456

[B48] Yan P. A. Su L. Chen J. Wu J. (2013). Heterologous expression and biochemical characterization of an endo-β-1,4-glucanase from *Thermobifida fusca*. *Biotechnol. Appl. Biochem.* 60 348–355. 10.1002/bab.1097 23631559

[B49] Yu L.-X. Gray B. N. Rutzke C. J. Walker L. P. Wilson D. B. Hanson M. R. (2007). Expression of thermostable microbial cellulases in the chloroplasts of nicotine-free tobacco. *J. Biotechnol.* 131 362–369. 10.1016/j.jbiotec.2007.07.942 17765995

[B50] Yuan J. S. Tiller K. H. Al-Ahmad H. Stewart N. R. Stewart C. N. Jr. (2008). Plants to power: bioenergy to fuel the future. *Trends Plant Sci.* 13 421–429. 10.1016/j.tplants.2008.06.001 18632303

[B51] Zhang S. Irwin D. C. Wilson D. B. (2000). Site-directed mutation of noncatalytic residues of *Thermobifida fusca* exocellulase Cel6B. *Eur. J. Biochem.* 267 3101–3115. 10.1046/j.1432-1327.2000.01315.x 10824094

[B52] Zhang S. Lao G. Wilson D. B. (1995). Characterization of a *Thermomonospora fusca* exocellulase. *Biochemistry* 34 3386–3395. 10.1021/bi00010a030 7880834

[B53] Ziegelhoffer T. Raasch J. A. Austin-Phillips S. (2001). Dramatic effects of truncation and sub-cellular targeting on the accumulation of recombinant microbial cellulase in tobacco. *Mol. Breed.* 8 147–158. 10.1023/A:1013338312948

[B54] Ziegelhoffer T. Will J. Austin-Phillips S. (1999). Expression of bacterial cellulase genes in transgenic alfalfa (*Medicago sativa* L.), potato (*Solanum tuberosum* L.) and tobacco (*Nicotiana tabacum* L.). *Mol. Breed.* 5 309–318. 10.1023/A:1009646830403

